# L-arginine enhances cell proliferation and reduces apoptosis in human endometrial RL95-2 cells

**DOI:** 10.1186/1477-7827-11-15

**Published:** 2013-02-26

**Authors:** Jonathan M Greene, Jean M Feugang, Kathryn E Pfeiffer, John V Stokes, Susan D Bowers, Peter L Ryan

**Affiliations:** 1 Department of Pathobiology and Population Medicine, Mississippi State University, Mississippi State, MS, USA; 2 Department of Animal and Dairy Sciences, Mississippi State University, Mississippi State, MS, USA; 3 Facility for Organismal and Cellular Imaging, Mississippi State University, Mississippi State, Mississippi, USA; 4 Department of Basic Sciences, Mississippi State University, Mississippi State, Mississippi, USA

**Keywords:** L-arginine, Uterus, Endometrium, Cell proliferation, Apoptosis

## Abstract

**Background:**

L-arginine is considered to be one of the most versatile amino acids due to the fact that it serves as a precursor for many important molecules in cellular physiology. When supplemented in the diet, L-arginine can increase the number of implantation sites in mice and rats, suggesting an effect at the level of the endometrium. To this end, this study determined the effect that L-arginine has on apoptosis and cell proliferation in human endometrial RL95-2 cells.

**Results:**

L-arginine at physiological (200 micromol/L) and supra-physiological (800 micromol/L) concentrations increased cell proliferation at days 2 and 4 post-treatment with a dose-dependent effect being observed on day 2. Additionally, inhibition of nitric oxide (NO) synthase and arginase, which are responsible for the conversion of L-arginine to NO and polyamines, respectively, reduced the proliferative effect of L-arginine. L-arginine also decreased the proportion of cells with TUNEL positive nuclei and increased the ratio of cells with healthy mitochondria compared to cells with a disrupted mitochondrial membrane potential, indicating that L-arginine prevents mitochondrial mediated apoptosis in endometrial RL95-2 cells. Furthermore, exposure to L-arginine did not affect total BAD protein expression; however, L-arginine increased the abundance of phosphorylated BAD protein.

**Conclusions:**

In summary, L-arginine added to the culture media at physiological (200 micromol/L) and supraphysiological concentrations (800 micromol/L) enhanced endometrial RL95-2 cell proliferation through mechanisms mediated by NO and polyamine biosynthesis. In addition, L-arginine reduced endometrial RL95-2 mitochondrial mediated apoptosis through increased phosphorylation of BAD protein.

## Background

L-arginine is considered to be a conditionally essential amino acid for healthy mature mammals but an essential amino acid for young developing mammals [1], suggesting a role for arginine in tissue growth. Most dietary sources of protein contain L-arginine; however, L-arginine is found in abundant quantities in high quality plant proteins (i.e. soy proteins), and daily intake of L-arginine for adult humans ranges from 3 to 6 g [2]. In addition to being incorporated into proteins and being involved in ammonia detoxification [3], L-arginine also serves as a precursor for many molecules that are important for cellular physiology, including proline, glutamate, creatine, nitric oxide (NO) and polyamines, making L-arginine one of the most versatile amino acids [4]. L-arginine is converted to NO through the action of NO synthase (NOS), while polyamines are generated through the conversion of L-arginine to ornithine via arginase [4]. Decarboxylation of ornithine by ornithine decarboxylase yields the first polyamine putrescine which serves as the precursor for the other naturally occurring polyamines spermidine and spermine through the action of spermidine synthase and spermine synthase, respectively [4].

Both polyamines and NO have vital roles in cellular processes and cell signaling. Nitric oxide and polyamines stimulate cell proliferation and have a positive effect on progression through the cell cycle [5-11]. Polyamines exert their cellular effect through their ability to bind nucleic acids and proteins [12] and have been demonstrated to promote an anti-apoptotic state in various cell lines [13]. Moreover, NO can stimulate PI3K/Akt-1 signaling pathways [14-16] which promote cell survival. The role of L-arginine and its metabolites in cell signaling has been studied extensively in ovine [9,17] and porcine [18] trophectoderm cells, with L-arginine enhancing cell proliferation through mammalian target of rapamycin (mTOR) related signaling pathways.

Wu et al. [19] reported an unusual abundance of L-arginine in porcine allantoic fluid, suggesting a role for this particular amino acid in fetoplacental nutrition. Moreover, dietary L-arginine supplementation has been demonstrated to enhance the reproductive performance of rats [20], pigs [21], and mice [22], and recently, we have shown that dietary L-arginine supplementation increased the number of implantation sites in mice [22], which suggests an effect of L-arginine at the level of the endometrium. The endometrium has the ability to catabolized L-arginine in numerous species, including sheep [23-25], pigs [26], mice [27], rats [20], and humans [28], due to the presence of NOS and/or arginase enzymes. Moreover, L-arginine has been reported to exist in human uterine lumen flushes with the greatest concentration observed during the proliferative phase [29], suggesting a possible involvement in endometrial epithelial cell proliferation.

In the current study we used the human endometrial epithelial carcinoma cell line, RL95-2, as a model for endometrial epithelial cells. The RL95-2 cell line expresses markers found on normal human endometrial epithelial cells such as progesterone receptors, estrogen receptors α and β [30], MUC1 [31], and E-cadherin [32,33]. Furthermore, the RL95-2 cell line has been used extensively as an *in vitro* model for studying the human endometrial epithelium [30,34-36]. To this end, the objective of this study was to examine the effect that L-arginine may have on endometrial cell proliferation and apoptosis using the established human endometrial epithelial cell line, RL95-2, as an *in vitro* model for epithelial cells of the human endometrium.

## Methods

### Cell culture

Human endometrial carcinoma cells (RL95-2; ATCC # CRL-1671) were acquired from the American Type Culture Collection (Rockville, MD). Cells were cultured in a humidified incubator containing 5% CO_2_ using a complete growth media comprised of DMEM:F12 media (ATCC, Rockville, MD) supplemented with 10% fetal bovine serum (FBS; Gibco, Grand Island, NY), 1% penicillin/streptomycin (Gibco, Grand Island, NY), and 0.005 mg/mL insulin (Sigma-Aldrich, St. Louis, MO) in order to obtain frozen stocks.

### Proliferation assay

RL95-2 cells were transferred to 96 well plates (80,000 cells per well) in growth media for a period of 24 h after which they were serum and L-arginine starved for an additional 24 hours in an L-arginine free media (RPMI-1640 SILAC, Sigma-Aldrich, St. Louis, MO). In the first experiment, cells were then treated (n = 3 wells per treatment) with either 0 μmol/L, 200 μmol/L (physiological), or 800 μmol/L L-arginine (Sigma-Aldrich, St. Louis, MO) in a serum-free environment. At two days post-treatment, cell proliferation was assessed for one plate of cells, and the media was replenished in the second plate of cells. Cell proliferation was then assessed in the second plate 4 days after the initial treatment. In the second experiment, cells were treated with 0 μmol/L, 200 μmol/L, or 800 μmol/L L-arginine with or without N-omega-hydroxy-nor-arginine (Nor-NOHA; Calbiochem-EMD4 Biosciences, Billerica, MA), a polyamine synthesis inhibitor, in a serum-free environment. The media was replenished on day 2 post-treatment, and cell proliferation was assessed on day 4 post-treatment. Additionally, a third experiment examined the role of NO biosynthesis in endometrial RL95-2 cell proliferation: cells were treated with either 0 μmol/L, 200 μmol/L, or 800 μmol/L L-arginine with or without 7-Nitroindazole (7-NI), a NOS inhibitor, in a serum-free environment. 7-NI was dissolved in ethanol, and all cells not exposed to 7-NI received an equal amount of ethanol. Cell proliferation was assessed according to procedures previously described by Kueng et al. [37]. Briefly, cells were washed in Dulbecco′s PBS (DPBS) and fixed in 3% glutaraldehyde for 15 min. Fixed cells were washed three times by submersion in de-ionized water and air dried, after which they were stained with crystal violet (0.1% in 20% methanol) for 20 min, followed by three washes with de-ionized water. Crystal violet was eluted using 10% glacial acetic acid, and the optical density was measured at 590 nm. All experiments were repeated independently three times.

### Detection of DNA fragmentation

RL95-2 cells were transferred to chamber slides (100,000 cells per chamber) in growth media for a period of 24 h, after which they were serum and L-arginine starved for an additional 24 hours in an L-arginine free media (RPMI-1640 SILAC). Cells were then treated (n = 1 chamber per treatment) with either 0 μmol/L, 200 μmol/L, or 800 μmol/L L-arginine in a serum-free environment for 24 hours. Cells were washed with DPBS and fixed in a solution of 4% paraformaldehyde in PBS for 60 min, washed with DPBS, and incubated with a permeabilization solution (0.1% Triton X-100 in 0.1% sodium citrate) for 2 min on ice followed by two washes with DPBS. DNA fragmentation was detected by incubating cells with a FITC-labeled terminal deoxynucleotidyl transferase dUTP nick end labeling (TUNEL) solution (Roche Applied Science, Indianapolis, IN) at 37°C in a humidified incubator. After 60 min, cells were washed three times with DPBS, the nucleus was counter-stained with DAPI (Santa Cruz Biotechnology, Santa Cruz, CA), and the slides where covered with a coverslip. TUNEL (ex. 490/20; em. 528/30) and DAPI (ex. 350/50; em. 457/50) staining were assessed using a Nikon Eclipse TE 2000-U fluorescence microscope. Three frames per chamber were acquired, and the proportion of cells that were TUNEL positive was counted. The entire experiment was repeated three independent times.

### Assessment of mitochondrial membrane potential (ΔΨm)

RL95-2 cells were seeded into 12-well plates (750,000 cells per well) and grown for 24 hours in growth media at which time they were serum and L-arginine starved for an additional 24 hours. Cells were then treated (n = 3 wells per treatment) with either 0 μmol/L, 200 μmol/L, or 800 μmol/L L-arginine in a serum-free environment for 24 hours, followed by incubation with 5,5^′^,6,6^′^-tetrachloro-1,1^′^,3,3^′^-tetraethylbenzimidazole-carbocyanide iodine (JC-1; Cayman Chemical, Ann Arbor, MI) for 30 minutes at 37°C. Loss of ΔΨm was determined using fluorescence microscopy (Nikon Eclipse TE 2000-U) and flow cytometry (Becton Dickinson FACSCalibur). Immediately, following incubation with JC-1, fluorescence microscopy was performed using a 490 nm excitation filter, with an orange emission indicating healthy ΔΨm which is due to a potential-dependent aggregation of JC-1 molecules in the mitochondria. In contrast, a loss of ΔΨm results in the monomeric form of JC-1 in the cytosol which produces a green emission. For quantification, JC-1 labeled cells were harvested using EDTA (0.02% in PBS) and analyzed by flow cytometry (3,000 cells per sample). Excitation was achieved with a 488 nm argon laser, and emission fluorescence was measured in the FL-1 (530/30 nm) and FL-2 (585/42 nm) channels to determine the proportion of cells with JC-1 monomers or JC-1 aggregates, respectively. From this analysis, the ratio of cells with JC-aggregates compared to cells with JC-1 monomers was determined. Flow cytometry analysis was repeated three independent times, and fluorescence microscopy was performed once to obtain representative images.

### Reverse transcriptase real time PCR

RL95-2 cells were transferred to culture dishes (1.0 × 10^6^ cells per dish) in growth media for a period of 24 h after which they were serum and L-arginine starved for an additional 24 hours in an L-arginine free media (RPMI-1640 SILAC). Cells were then treated (n = 3 culture dishes per treatment) with either 0 μmol/L, 200 μmol/L, or 800 μmol/L L-arginine in a serum-free environment. After 24 hours, cells were washed in cold DPBS, trypsinized, and stored as pellets at −80°C. Total RNA was isolated (High Pure RNA Isolation kit; Roche Applied Science, Indianapolis, IN), quantified (Nanodrop 1000; Thermo Scientific, Wilminton, DE), and reverse transcribed into cDNA (Transcriptor First Strand cDNA Synthesis; Roche Applied Science, Indianapolis, IN) using 500 ng of total RNA.

For gene expression analysis, NCBI Primer BLAST was used to design primers for BAX, BCL2, and 18s rRNA (Table 1). Real-time PCR (Stratagene MX3005P; Agilent Technologies, Inc, Santa Clara, CA) was performed using 0.5 μL of cDNA, a final concentration of 0.5 μM of each primer, and SYBR Green I Master Mix (Roche Applied Science, Indianapolis, IN). The PCR conditions were the following: 5 min at 95°C; 40 cycles of 30 sec at 95°C; 30 sec at the optimal annealing temperature (Table 1); 30 sec at 72°C. Relative gene expression was calculated using the 2^-ΔΔCT^ method [38]. The entire experiment was repeated three independent times.

**Table 1:** Primer sequences and characteristics

**Gene**	**GenBank Acc. #**	**Primer sequences (5**^′^-3^′^)	**Annealing T (°C)**	**Amplicon size (bp)**
BAX	NM_004324.3	S: TCTGACGGCAACTTCAACTG	54.5	155
		AS: TTGAGGAGTCTCACCCAACC		
BCL2	NM_000633.2	S: CGTCAACCGGGAGATGTCGCC	62.0	132
		AS: CTGGGGCCGTACAGTTCCACA		
18s rRNA	X03205.1	S: AAACGGCTACCACATCCAAG	56.0	188
		AS: CCTCCAATGGATCCTCGTTA		

*S*, Sense; *AS*, Anti-sense.

### In cell ELISA and Western-immunoblot detection of BCL2, BAX, BAD, and p-BAD proteins

In a 96-well plate, RL95-2 cells were cultured (80,000 cells per well) in growth media for a period of 24 h, after which they were serum and L-arginine starved for an additional 24 hours in an L-arginine free media (RPMI-1640 SILAC). Cells were then treated (n = 3 wells per treatment) with either 0 μmol/L, 200 μmol/L, or 800 μmol/L L-arginine in a serum-free environment. After 24 hours, cells were fixed with paraformaldehyde (4% in PBS). BCL2, BAX, BAD, and p-BAD expression was assessed using the Pierce Colormetric In-Cell ELISA kit (Thermo Scientific, Waltham, MA) as per the manufacturer’s instructions. BCL2, BAX, BAD, and p-BAD measurements were obtained and normalized to cell number using the Janus Green Whole-Cell stain supplied with the kit. The entire experiment was repeated three independent times. Total protein was isolated from frozen-thawed RL95-2 cells using complete RIPA buffer (Santa Cruz Biotech Inc, Santa Cruz, CA). Isolated protein was resolved onto an SDS PAGE gel and transferred to a PVDF membrane. The WesternBreeze Chromogenic kit was utilized for immunodection as per the manufacturer’s instructions (Life Technologies, Inc. Grand Island, NY). Primary antibody concentration for western-immunoblotting were the following: BCL2: 0.004 μg/μL; BAX: 0.004 μg/μL; p-BAD: 0.008 μg/μL; and BAD: 0.001 μg/μL. BCL2 (sc-130307), BAD (sc-8044) and serine-136 phosphorylated BAD (p-BAD; sc-12969) primary antibodies were obtained from Santa Cruz Biotechnolgy, Inc. BAX primary antibody (B3428) was obtained from Sigma-Aldrich, Inc.

### Statistical analysis

The Shapiro-Wilk test was utilized to test the data for normal distribution. All data were normally distributed except for cell-proliferation and JC-1 data. Normally distributed data were analyzed using one-way or two-way ANOVA, when appropriate, followed by Fisher’s LSD test for pairwise comparison. Data that tested to be non-parametric were analyzed by Friedman’s one-way or two-way non-parametric ANOVA, when appropriate, followed by Tukey’s HSD test for pairwise comparison. The threshold of significance was fixed at P < 0.05. Data are presented as least square means ± standard error of the mean (SEM).

## Results

### Effect of L-arginine on endometrial RL95-2 cell proliferation

**Figure 1:**
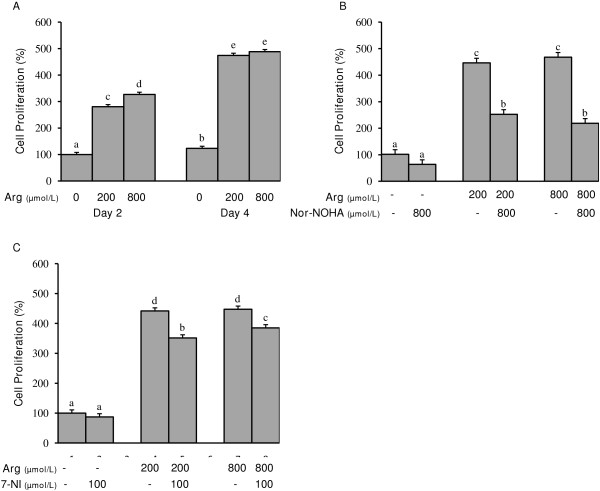
**A) Effect of L-arginine on human endometrial RL95-2 cell proliferation; B) Effect of L-arginine and the arginase inhibitor Nor-NOHA on human endometrial RL95-2 cell proliferation; C) Effect of L-arginine and the NOS inhibitor 7-NI on human endometrial RL95-2 cell proliferation.** Each bar represents the mean ± S.E.M.; a,b,c,d,e means without common letters are significantly different (P < 0.05).

The presence of L-arginine at physiological (200 μmol/L) and supraphysiological (800 μmol/L) concentrations increased (P < 0.05) endometrial RL95-2 cell proliferation at days 2 and 4 post-treatment with proliferation being increased by approximately 4-fold on day 4 (Figure 1A). Additionally, a dose dependent effect of L-arginine on endometrial RL95-2 cell proliferation was observed on day 2 post-treatment at which time cell proliferation was greater (P < 0.05) for cells treated with 800 μmol/L L-arginine compared to those exposed to 200 μmol/L.

### Inhibitory Effect of nor-NOHA on endometrial RL95-2 cell proliferation

To test whether polyamines, L-arginine metabolites, are responsible for L-arginine’s effect on cell proliferation, cells were exposed to L-arginine and the arginase inhibitor nor-NOHA. As in experiment one, the addition of L-arginine (200 μmol/L and 800 μmol/L) increased (P < 0.05) endometrial RL95-2 cell proliferation, but this effect was reduced 2-fold (P < 0.05) with the addition of 800 μmol/L nor-NOHA (Figure 1B).

### Inhibitory effect of 7-NI on endometrial RL95-2 cell proliferation

Cells were exposed to L-arginine and the NOS inhibitor 7-NI to determine if L-arginine enhances endometrial RL95-2 cell proliferation through NO biosynthesis. Again, L-arginine (200 μmol/L and 800 μmol/L) increased (P < 0.05) endometrial RL95-2 cell proliferation, and this effect on cell proliferation was reduced (P < 0.05) with the addition of 100 μmol/L of 7-NI (Figure 1C).

### Effect of L-arginine on endometrial RL95-2 cell apoptosis

**Figure 2:**
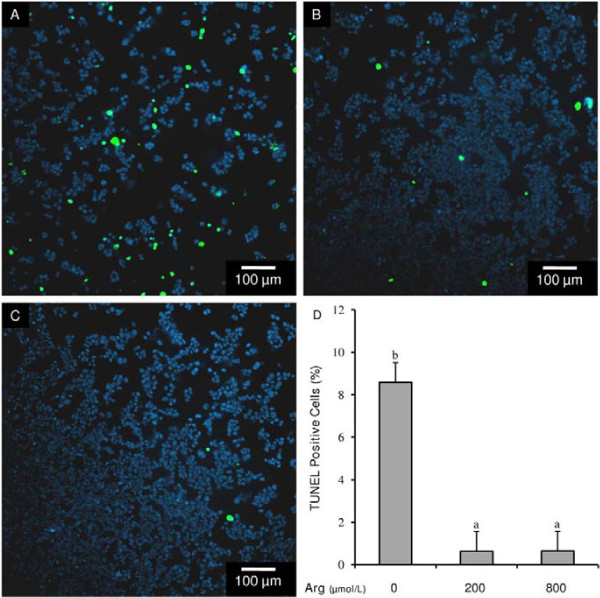
**Effect of L-arginine on human endometrial RL95-2 cell apoptosis as assessed by TUNEL assay and fluorescent microscopy.** Representative micrographs of cells exposed to (**A**) 0 μmol/L, (**B**) 200 μmol/L, or (**C**) 800 μmol/L L-arginine. Cells staining green are TUNEL positive cells experiencing DNA fragmentation. (**D**) Percentage of cells staining positive for TUNEL after exposure to either 0 μmol/L, 200 μmol/L, 800 μmol/L L-arginine. Each bar represents the mean ± S.E.M.; a,b means without common letters are significantly different (P < 0.05).

Because of the inverse relationship that exists between cell proliferation and apoptosis [39], we sought to determine if L-arginine’s positive effect on cell proliferation was associated with a concomitant decrease in apoptosis. The addition of L-arginine (200 μmol/L and 800 μmol/L) decreased (P < 0.05) the proportion of cells that stained positive for TUNEL by approximately 13-fold, indicating a reduction in DNA fragmentation and, thus, apoptosis in the presence of L-arginine (Figure 2A-D).

### Effect of L-arginine on mitochondiral membrane potential (ΔΨm)

**Figure 3:**
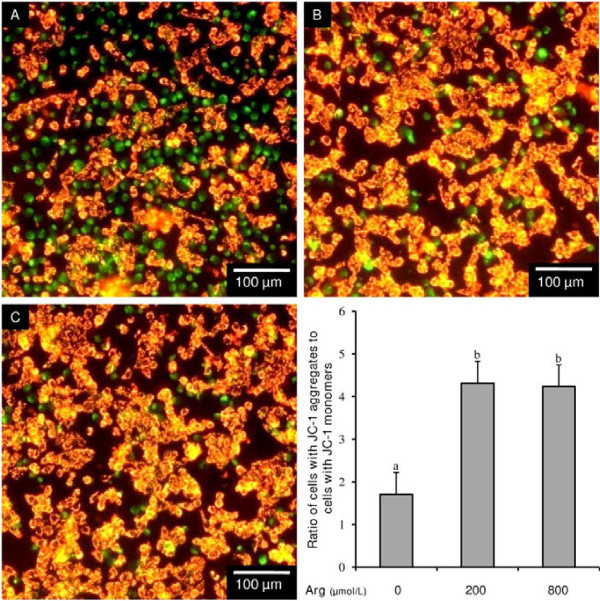
**Effect of L-arginine on human endometrial RL95-2 cell mitochondrial membrane potential (ΔΨm) as assessed by JC-1 staining.** Representative micrographs of cells exposed to (**A**) 0 μmol/L, (**B**) 200 μmol/L, or (**C**) 800 μmol/L L-arginine. Cells staining green (JC-1 monomers) are cells with a disrupted ΔΨm, while cells staining orange (JC-1 aggregates) have a healthy ΔΨm. (**D**) Ratio of cells with JC-1 aggregates to cells with JC-1 monomers as assessed by flow cytometry. Each bar represents the mean ± S.E.M.; a,b means without common letters are significantly different (P < 0.05).

Fluorescence microscopy analysis of JC-1 stained endometrial RL95-2 cells revealed that the presence of L-arginine (200 μmol/L and 800 μmol/L) increased the proportion of cells with healthy ΔΨm, as indicated by more cells yielding an orange emission upon excitation (Figure 3A-C). Furthermore, flow cytometry revealed that the addition of L-arginine to the culture media increased (P < 0.05) the ratio of cells with JC-1 aggregates compared to cells with JC-1 monomers by approximately 2.5-fold (Figure 3D), indicating that L-arginine reduces mitochondrial membrane potential disruption in endometrial RL95-2 cells.

### Effect of L-arginine on BAX and BCL2 gene and protein expression

**Figure 4:**
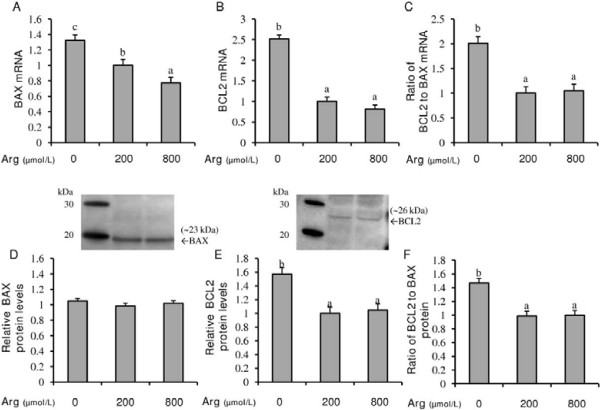
**Effect of L-arginine on (A) BAX mRNA, (B) BCL2 mRNA, (C) ratio of BCL2 to BAX mRNA, (D) BAX protein, (E) BCL2 protein, and (F) the ratio of BCL2 to BAX protein in human endometrial RL95-2 cells.** Western-immunoblotting of RL95-2 total cell protein for BAX (**D**) and BCL2 (**E**) yielded proteins of approximately 23 kDa and 26 kDa, respectively. Each bar represents the mean ± S.E.M.; a,b means without common letters are significantly different (P < 0.05).

The presence of L-arginine at physiological (200 μmol/L) and supraphysiological (800 μmol/L) concentrations dose-dependently reduced (P < 0.05) the amount of *BAX* mRNA expression, with endometrial RL95-2 cells exposed to 800 μmol/L L-arginine expressing the least (P < 0.05) amount of *BAX* mRNA (Figure 4A). Interestingly, cells exposed to L-arginine also expressed less (P < 0.05) *BCL2* mRNA, and had a lower (P < 0.05) *BCL2* to *BAX* mRNA ratio (Figure 4B and C). Exposure to L-arginine resulted in a *BCL2* to *BAX* mRNA ratio of approximately one, while cells not exposed to L-arginine exhibited a ratio of two. L-arginine at physiological (200 μmol/L) and supraphysiological (800 μmol/L) concentrations had no effect on BAX protein expression (Figure 4D); however, in cells that were not exposed to L-arginine, BCL2 protein levels were elevated (P < 0.05; Figure 4E). Additionally, cells exposed to L-arginine had a lower (P < 0.05) BCL2 to BAX protein ratio compared to cells not exposed to L-arginine (Figure 4F).

### Effect of L-arginine on phosphorylation of BAD protein

**Figure 5:**
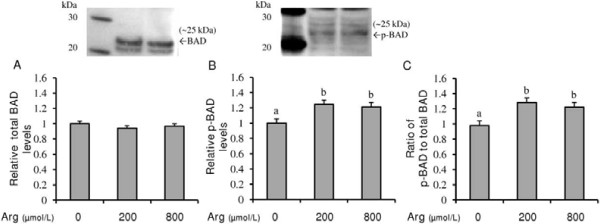
**Effect of L-arginine on (A) total BAD protein, (B) phosphorylated BAD (p-BAD) protein, and (C) the ratio of p-BAD protein to total BAD protein in human endometrial RL95-2 cells.** Western-immunoblotting of RL95-2 total cell protein for BAD (**A**) and p-BAD (**B**) yielded proteins of approximately 25 kDa. Each bar represents the mean ± S.E.M.; a,b means without common letters are significantly different (P < 0.05).

Because L-arginine did not increase the BCL2 to BAX mRNA and protein ratio, an alternate mechanism for L-arginine’s promotion of cell survival and prevention of apoptosis was investigated. To this end, endometrial RL95-2 cells were exposed to 0, 200, or 800 μmol/L of L-arginine to determine total and phosphorylated forms of BAD, which is a promoter of mitochondrial mediated apoptosis when not phosphorylated [40]. L-arginine at 200 and 800 μmol/L did not affect the relative levels of total BAD protein in RL95-2 cells (Figure 5A). However, the addition of L-arginine did increase (P < 0.05) the relative levels of phosphorylated (Ser-136) BAD protein and, thus, the ratio of phosphorylated BAD protein to total BAD protein in endometrial RL95-2 cells (Figure 5B and C).

## Discussion

L-arginine is a versatile amino acid, serving as a precursor for many molecules including NO and polyamines [4]. The plasma concentration of L-arginine has been reported to be around 200 μmol/L in humans during the fed state [4,41]. Therefore, we sought to determine the effect of L-arginine on endometrial RL95-2 cells at physiological (200 μmol/L) and supraphysiological (800 μmol/L) concentrations. The presence of NOS and/or arginase enzymes in the endometrium of many species indicates the ability of the endometrium to catabolize L-arginine [20,23-28]. In females, NO is produced in the endometrium [42] and is involved in embryo implantation and development [43-45]. Polyamines are also produced by the endometrium [46,47] and have been shown to be important for embryo implantation, as inhibition of polyamine synthesis reduced pregnancy rates in mice [46].

L-arginine has been reported to be present in the uterine flushes of sheep [48], cows [49], rats [50], and humans [29], with concentrations in human uterine flushes ranging from 220 μmol/L to 330 μmol/L depending upon the phase of the menstrual cycle [29]. Additional work has revealed that mRNA of the L-arginine transporters SLC7A1, SLC7A2, and SLC7A3 are present in ovine uterine luminal epithelial [51]. Furthermore, the positive influence that L-argnine has on cell signaling, proliferation, hypertrophy, hyperplasia, and migration of ovine trophectoderm cells [9,17] suggests that L-arginine is transported into the uterine lumen to support growth and development of the peri-implantation embryo.

In addition to supporting the peri-implantation embryo, L-arginine may also have a direct effect on the uterine luminal epithelium. Proliferation of the endometrium has been implicated as a vital process which provides an optimal environment for embryo adhesion and implantation [52], and this argument is further supported by the observation that increasing endometrial thickness is associated with improved implantation rates in humans [53-55]. Interestingly, the uterine lumen concentration of L-arginine is greatest during the proliferative phase of the menstrual cycle [29], suggesting that L-arginine may have a role in the proliferation of the endometrial epithelium which must regenerate following menstruation. L-arginine and its metabolites, NO and polyamines, have a dual role in cell proliferation and apoptosis. In some cell types, L-arginine, NO, and polyamines stimulate cell proliferation and reduce apoptosis [8,9,56,57], yet they inhibit cell proliferation and promote apoptosis in others [58-61]. Results from the current study indicate that L-argrinine enhances endometrial RL95-2 cell proliferation at physiological and supraphysiological concentrations. Moreover, Nor-NOHA, an arginase inhibitor, and 7-NI, an NOS inhibitor, reduced the positive effect that L-arginine had on endometrial RL95-2 cell proliferation. Conversion of L-arginine to ornithine, via arginase, is the first enzymatic process involved in polyamine synthesis [4]. Likewise, NOS is responsible for converting L-arginine to NO [4]. Together, the inhibitory effect that Nor-NOHA and 7-NI exhibited in the presence of L-arginine indicates that L-arginine enhances endometrial RL95-2 cell proliferation through polyamine and NO mediated pathways, which both have a positive influence on cell proliferation [5-11].

Cell proliferation is often inversely related to apoptosis [39,62-64], and a reduction in apoptosis is a contributing factor in the enhancement of cell proliferation [65]. Therefore, we hypothesized that the enhancement of cell proliferation in the presence of L-arginine would be associated with decreased endometrial RL95-2 cell apoptosis. Apoptosis in the endometrium is a key feature of the human menstrual cycle and aids in maintaining endometrial homeostasis by eliminating cells from the functionalis layer during the late secretory phase [66]. In the functionalis layer of the endometrium, apoptosis exhibits a cyclic pattern with the least amount being observed during the proliferative phase followed by an increase during the secretory phase and the maximum being observed during menstruation [67,68]. The exposure of endometrial RL95-2 cells to physiological and supraphysiological concentrations of L-arginine reduced the proportion of cells that exhibited DNA fragmentation as assessed by TUNEL assay. Activation of endonucleases [69] and the subsequent DNA fragmentation [70] are considered to be hallmark characteristics of cells undergoing apoptosis. To this end, the current results demonstrate that the presence of L-arginine reduces the proportion of endometrial RL95-2 cells experiencing apoptosis. Apoptosis can occur through either receptor-ligand mediated pathways or mitochondrial mediated pathways, with both resulting in DNA fragmentation [71]. Receptor-ligand mediated apoptosis requires an external signal, while mitochondrial mediated apoptosis occurs through the disruption of the mitochondrial membrane [71]. As the presence or absence of L-arginine would represent an intracellular event rather than receptor mediated extracellular signaling, we hypothesized that L-arginine’s prevention of apoptosis in endometrial RL95-2 cells is mediated through the mitochondria. The presence of L-arginine in the culture media increased the ratio of cells with a healthy mitochondrial membrane compared to cells with an altered mitochondrial membrane potential. Thus, the current study indicates that L-arginine reduces the incidence of endometrial RL95-2 cell apoptosis by preventing the disruption of mitochondrial membrane potential, suggesting a role for L-arginine in the regulation of endometrial epithelial apoptosis.

Mitochondrial membrane potential is highly influenced by proteins that belong to the BCL2 family [72]. The pro-apoptotic protein BAX and the anti-apoptotic protein BCL2 are often studied together as indicators of apoptosis. In healthy cells, a balance exists in which BCL2 is normally found imbedded in the mitochondrial membrane [73]. Under apoptotic conditions, activated BAX will embed in the mitochondrial membrane with BCL2 and disrupt the mitochondrial membrane potential [73]. Accordingly, we examined if L-arginine’s prevention of apoptosis is through a BCL2 and BAX mediated event. Interestingly, the presence of L-arginine did not increase the ratio of BCL2 to BAX in endometrial RL95-2 cells. In fact, the BCL2 to BAX mRNA and protein ratios were higher in endometrial RL95-2 cells not exposed to L-arginine which were undergoing apoptosis through a mitochondrial mediated pathway. Despite the anti-apoptotic properties of BCL2, upregulation of BCL2 mRNA and protein has been reported in cells undergoing apoptosis [74]. Moreover, increased expression of BCL2 protein can lead to disruption of mitochondrial membrane potential [75], as caspases can cleave BCL2 into a BAX-like molecule which can serve as a latent pro-apoptotic stimuli in apoptotic cells [76].

Because exposure to L-arginine did not increase the ratio of BCL2 to BAX, we hypothesized that L-arginine might decrease endometrial RL95-2 cell apoptosis through an alternative mechanism. In addition to BCL2 and BAX, BAD is another member of the BCL2 family of proteins that affects mitochondrial membrane potential. The presence of L-arginine in the culture media did not affect the levels of total BAD. However, L-arginine increased p-BAD (Ser-136) levels in endometrial RL95-2 cells and increased the ratio of p-BAD to BAD, indicating that L-arginine enhances the phosphorylation of BAD protein at serine residue 136 in endometrial RL95-2 cells. When BAD is phosphorylated at either serine residue 112 (Ser-112) or 136 (Ser-136), it is bound by 14-3-3 and sequestered in the cytosol [40]. In contrast, non-phosphorylated BAD interacts with BCL2 and BCL-XL embedded in the mitochondrial membrane and inhibits their anti-apoptotic properties [77,78] and causes release of cytochrome C [79]. In this regard, L-arginine reduces mitochondrial membrane disruption and, thus, apoptosis through phosphorylation of BAD in endometrial RL95-2 cells. BAD protein is phosphorylated at serine residue 136 through the kinase activity of PI3K-dependent Akt-1 [80]. L-arginine increases phosphorylation, and therefore the activity, of Akt-1 in ovine trophectoderm cells [17]. Moreover, NO can stimulate phosphorylation of Akt-1 [14-16], and Akt-1 phosphorylation is also enhanced in cells with elevated expression of ornithine decarboxylase [81], the enzyme responsible for converting ornithine to first polyamine putrescine. Thus, it is likely that the presence of L-arginine in the culture media increased p-BAD levels in endometrial RL95-2 cells by influencing Akt-1 phosphorylation through the action of polyamines and/or NO.

## Conclusions

In summary, L-arginine added to the culture media at physiological (200 μmol/L) and supraphysiological concentrations (800 μmol/L) enhanced endometrial RL95-2 cell proliferation through mechanisms mediated by NO and polyamine biosynthesis and by reducing endometrial RL95-2 cell apoptosis through the phosphorylation of BAD protein. Cell proliferation is an important process in the human endometrium, as the endometrial epithelium must regenerate following the losses experienced during menstruation in preparation for the attachment and implantation of a potential embryo. Accordingly, the findings of the present study demonstrate a role for L-arginine in the regulation of endometrial growth and apoptosis. Moreover, a supraphysiological concentration of L-arginine had no negative effects on the parameters measured, revealing a possible beneficial effect of dietary L-arginine supplementation on endometrial growth.

## Competing interests

The authors declare that they have no competing interests.

## Authors’ contributions

JMG, JMF, and PLR conceived the study and participated in the design of the study. JMG, JMF, KEP, JVS, and SDB performed the experiments. JMG conducted the statistical analysis. JMG, JMF, and PLR interpreted the data, and JMG drafted the manuscript. All authors read and approved the final manuscript.
